# Clinical outcomes of two revision strategies for failed total disc replacements

**DOI:** 10.1007/s00586-012-2354-4

**Published:** 2012-05-11

**Authors:** Ilona Punt, Paul Willems, Steven Kurtz, Lodewijk van Rhijn, André van Ooij

**Affiliations:** 1 Department of Orthopaedic Surgery, Maastricht University Medical Center, P.O. Box 5800, 6202 AZ Maastricht, The Netherlands; 2 Research School CAPHRI, Maastricht University, P.O. Box 616, 6200 MD Maastricht, The Netherlands; 3 Drexel University, Philadelphia, PA USA; 4 Department of Orthopaedic Surgery, Viecuri Medical Centre, P.O. Box 1926, 5900 BX Venlo, The Netherlands

**Keywords:** Total disc replacement, Spinal fusion, Revision surgery, VAS, Oswestry

## Abstract

**Purpose:**

To compare mid-term clinical outcomes of two revision strategies for patients with failed SB Charité III total disc replacements (TDRs).

**Methods:**

Eighteen patients with a failed TDR underwent posterolateral instrumented fusion (fusion group); in 21 patients, the TDR was removed and the intervertebral defect was filled with a bone strut graft, followed by an instrumented posterolateral fusion (removal group). Visual analogue scale (VAS) for pain and Oswestry Disability Index (ODI) were completed pre- and post-revision surgery. Intra- and post-operative complications of both revision strategies were assessed.

**Results:**

Mean follow-up was 3.7 years (range 1.0–6.4) in the removal group and 4.4 years (range 0.7–11.0) in the fusion group. Although the removal group showed a significantly lower VAS and ODI score post-revision surgery as compared to preoperative (*P* < 0.01 and *P* = 0.01, respectively), no significant differences were found between the removal and fusion groups before and after revision surgery in VAS and ODI. A clinical relevant improvement in VAS and ODI was found in 47 and 21 % respectively in the removal group, and in 22 and 27 % respectively in the fusion group. Substantial complications were observed only in the removal group.

**Conclusions:**

Both procedures showed improvement clinically. There were no significant additional benefits of removing the TDR as compared to fusion alone at mid-term follow-up. The clinical decision to remove the TDR should be carefully weighed up against potential risks and complications of this procedure.

## Introduction

Lumbar total disc replacement (TDR) is increasingly used in the surgical treatment of degenerative disc disease (DDD). TDR aims to remove the pain source while preserving vertebral motion at the degenerative operated level(s) to prevent the development of adjacent segment degeneration [1–6]. The debate whether TDR is more effective than lumbar spinal fusion in treating DDD is still going on [5, 7–12]. Recently, a prospective randomized study showed that there are no differences in safety and clinical outcomes after TDR as compared to spinal fusion, at a follow-up period of 5 years [8]. It was further reported that between 2 and 5-years follow-up, only in the TDR group device failures had been observed [8].

Potential complications after TDR are recurrent back and leg pain, caused by facet joint degeneration, subsidence, polyethylene wear, migration and adjacent segment degeneration [5, 7, 10, 13]. This warrants the need for surgical revision strategies [14]. In a recent systematic review of Eerenbeemt et al. [11], an overall revision surgery rate ranging from 3.7 to 11.4 % was found after TDR. An important question we should ask ourselves is: Will revision surgery be beneficial, and if so, what would be the best revision strategy for failed TDR? In a previous study, we reported short-term results of two revision strategies with a follow-up of 1 year, showing that TDR removal gave better results than posterolateral instrumented fusion alone [15]. Larger groups and longer follow-up were thought to be necessary to support possible advantages of TDR removal surgery.

The purpose of this study was to compare the clinical mid-term results of these two revision strategies for patients with a failed SB Charité III lumbar disc prosthesis. Posterolateral instrumented fusion alone was compared with TDR removal combined with anterior lumbar interbody fusion followed by posterolateral instrumented fusion.

## Materials and methods

### Patients

Ninety patients with a SB Charité III TDR (Waldemar Link, Germany; DePuy Spine, Raynham, MA, USA) were seen in the outpatient clinic. For all these patients TDR implantation had been performed elsewhere. After evaluation, in 39 patients one or more revision surgeries were performed. Indications for revision surgery were recurrent back and leg pain, and the presence of a TDR-related pathology such as facet joint degeneration, subsidence and migration as observed on plain radiographs, CT-scan and/or MRI (Table 1). Adjacent disc degeneration was observed in 13 patients; however, it is uncertain whether this is caused as a consequence of the TDR.

**Table 1:** Summary of patient and clinical variables for TDR removal and fusion only group

	TDR removal (*n* = 21)	Fusion only (*n* = 18)	*P* value
Sex (male:female)	15:6	10:8	0.31
Mean age insertion TDR	43.4 (range 32–56)	40.7 (range 30–63)	0.11
Mean time in situ TDR	9.1 (range 3.1–16.0)	7.2 (range 1.7–14.8)	0.20
Operated levels			
1 level			0.90
L2–L3	0	1	
L3–L4	2	0	
L4–L5	10	4	
L5–S1	4	9	
2 level			
L4–L5, L5–S1	4	4	
3 level			
L2–L3, L4–L5,L5–S1	1	0	
Complications			
Subsidence	8	9	
Migration	2	6	
Facet joint degeneration	10	14	
Breakage metal wire	2	4	
Osteolysis	0	1	
Adjacent disc degeneration	5	8	

**Fig. 1:**
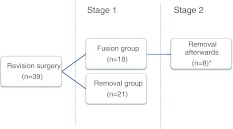
Overview of the patients who underwent revision surgery after TDR implantation. In these eight patients, pre- as well as post-revision surgery data were available for the fusion and removal revision surgeries (*asterisk*)

In 21 patients, the TDR was removed and after clearing of periprosthetic fibrous tissue and sclerotic bone, the intervertebral defect was filled with a bone strut graft. In addition, an instrumented posterolateral fusion was performed [16] (removal group). In the 18 other patients, posterolateral instrumented fusion without removal was performed (fusion group). The surgical technique of both surgeries was described in detail by de Maat et al. [16]. Because of persisting pain, in 8 of these 18 patients TDR removal was performed several years later as a second stage revision surgery (range 1.5–7.5 years). For these eight patients, data were available before and after fusion (stage 1) as well as before and after removal of the TDR (stage 2). An overview of the included patients is shown in Fig. 1.

### Clinical outcome measurements and complications

For all patients clinical evaluations were available pre- and post-revision surgery. These evaluations included a 10-point visual analogue scale (VAS) for pain and Oswestry Disability Index (ODI, 0–100 points) questionnaire. According to the FDA-criteria, clinical success was defined as a ≥25 % improvement in ODI between pre- and post-revision surgery [1]. Similarly for the VAS pain score, a ≥25 % improvement was considered as clinically successful. In addition, intra- and post-operative complications of both revision strategies were assessed.

### Statistical analysis

Analyses were performed using SPSS 16. Non parametric tests, i.e. Mann–Whitney and Wilcoxon, were used to test the mean. The mean values were given along ±standard error of the mean (SEM); *P* values <0.05 were considered statistically significant.

## Results

There were no significant differences between the removal group (*n* = 21) and fusion group (*n* = 18) with respect to sex, age at insertion of the TDR, mean time in situ of the TDR and number of operated levels (Table 1).

The mean follow-up in the removal group was 3.7 years (range 1.0–6.4 years) and 4.4 years (range 0.7–11.0 years) in the fusion group (*P* = 0.82).

### VAS pain scores

**Fig. 2:**
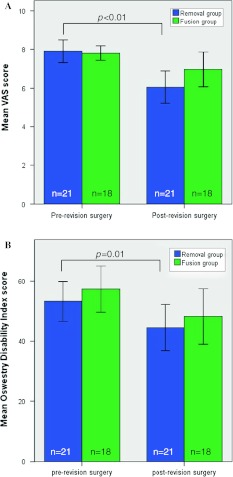
**a** Mean VAS scores for both groups pre- and post-revision surgery. **b** Mean Oswestry Disability Index for both groups during pre- and post-revision surgery. The *error bars* represent standard error of the mean

The mean ± SEM (standard error of the mean) pre-revision surgery VAS score was 7.9 ± 0.3 in the removal group and 7.8 ± 0.2 in the fusion group (*P* = 0.33). Post-revision VAS scores were 6.0 ± 0.4 and 7.0 ± 0.4 in the removal group and in the fusion group, respectively (*P* = 0.09). In both groups, a substantially lower VAS score was observed after revision surgery. Only in the removal group, there was a significant decrease in VAS score at post-revision surgery compared to pre-revision surgery (*P* < 0.01) (Fig. 2a).

**Fig. 3:**
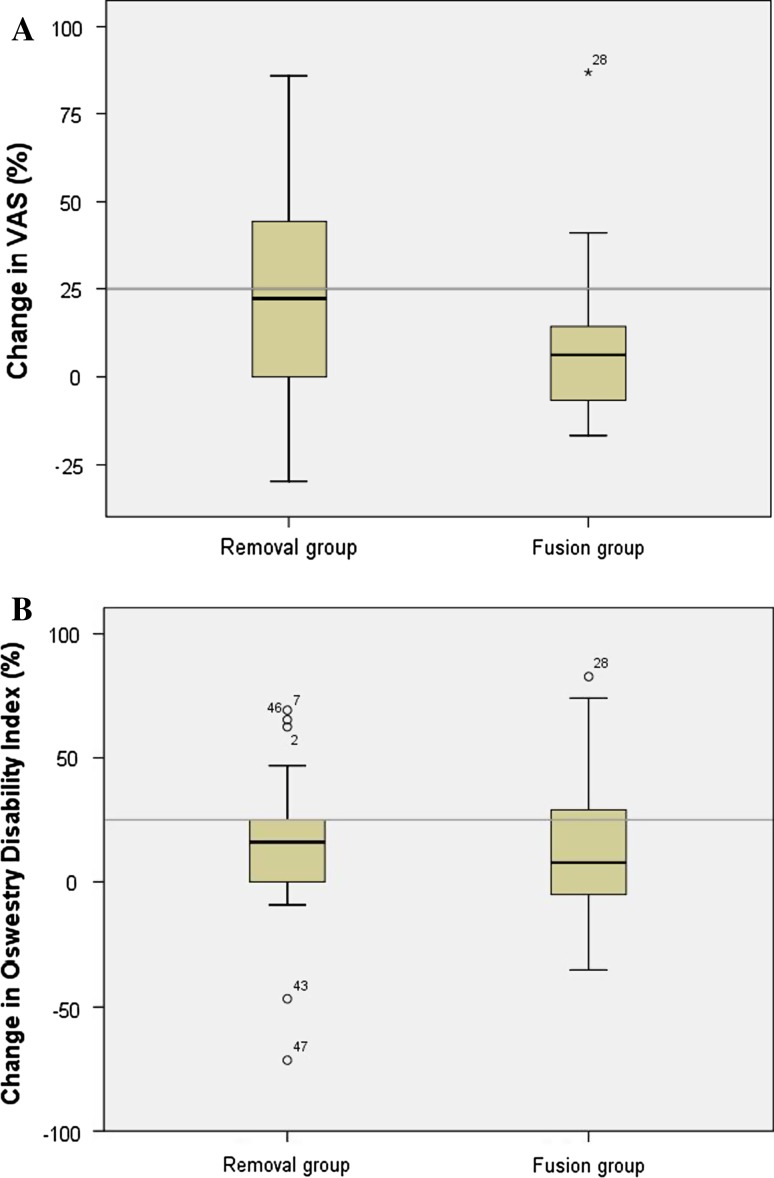
Box plot with **a** percentage change in VAS score in both revision strategy groups during pre- and post-revision surgery, **b** percentage change in ODI score in both revision strategy groups during pre- and post-revision surgery. The *line* represents a clinical success rate of 25 %. The *error bars* represent the upper and lower quartiles

The percentage of improvement after revision surgery in both groups is shown in Fig. 3a. According to the abovementioned FDA-criteria, in which an improvement of ≥25 % was considered to be clinically successful, 10 out of 21 patients (47.6 %) in the removal group and 4 out of 18 patients (22.2 %) in the fusion group were clinically improved (*P* = 0.14).

### Oswestry Disability Index

The mean pre- and post-revision surgery ODI scores were similar between the removal and fusion groups (*P* = 0.57, *P* = 0.61, respectively). The ODI in the removal group improved from 53.2 (±3.3) to 44.5 (±3.8) (*P* = 0.01), and in the fusion group from 57.3 (±3.9) to 48.2 (±4.6) (*P* = 0.06) (Fig. 2b).

The percentage of improvement after revision surgery in both groups is shown in Fig. 3b. A clinically relevant improvement of >25 % was present in 4 out of 21 patients (21.1 %) in the removal group and in 5 out of 18 patients (27.8 %) in the fusion group were clinically improved (*P* = 0.69).

### Second stage revision surgery

In the fusion group, the eight patients with persisting symptoms who underwent TDR removal at a later time-point as a second stage revision surgery had a mean follow-up period of 3.1 years (range 0.7–7.3) after fusion, while the other 10 patients had a mean follow-up of 5.6 years (range 1.8–11.0, *P* = 0.01). From the abovementioned eight patients, a mean follow-up of 3.1 years (range 1.4–5.0) was available after their second stage revision surgery, TDR removal.

**Fig. 4:**
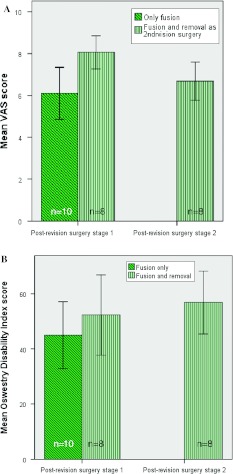
**a** Mean VAS scores for the fusion subgroups. **b** Mean Oswestry Disability Index scores for the fusion subgroups. Of the 18 patients, eight patients underwent TDR removal as a second revision surgery. The *error bars* represent standard error of the mean

In the fusion group, there was a significant difference in post-revision surgery VAS score between the patients who underwent TDR removal at a later time-point (*n* = 8) and those who did not (*n* = 10) (*P* < 0.01). The patient group who underwent removal as a second stage revision surgery (*n* = 8) had a decreased VAS score of 8.1 ± 0.4 to 6.7 ± 0.5 in time (*P* = 0.06) (Fig. 4a).

Patients in the fusion group who would undergo removal as a second stage revision had a similar ODI score as those who would not (*P* = 0.60 post-revision surgery stage 1). The patient group who underwent removal as a second revision surgery (*n* = 8) had a ODI score of 52.3 ± 7.3 and 56.8 ± 5.7 after stage 1 and stage 2 revision surgery, respectively (Fig. 4b).

### Complications

An overview of the intra- and post-operative complications from both revision procedures is shown in Table 2. Intra-operatively, no complications were seen in the fusion group, whereas in the removal group different types of vessel bleeding (*n* = 6), a small colon lesion (*n* = 1) and decreased sensitivity of the groyne (*n* = 2) were observed. In one patient TDR removal was planned, however, due to an intra-operative rupture of the small intestine this procedure was abandoned and only posterior fusion was performed. This patient was thus included in the fusion group. In one patient a lesion of the ureter occurred intra-operatively which necessitated resection of the left kidney at a second stage. In the fusion only group two patients developed a pseudo-arthrosis.

**Table 2:** Intra- and post-operative complications resulting from both revision strategies

	TDR removal (*n* = 21)	Fusion only (*n* = 18)
Intra-operative	Left common iliac artery lesion (*n* = 1)	
	Left common iliac vein lesions (*n* = 2), one resulting in a deep venous thrombosis of the left leg	
	Bleeding ascending lumbar vein (*n* = 1)	
	Pronounced bleeding intervertebral defect (*n* = 1)	
	Major blood loss (5,100 cc) (*n* = 1)	
	Small colon lesion (*n* = 1)	
	Decreased sensitivity in the left groyne (*n* = 2)	
Post-operative: 0–3 years	Resection left kidney after ureter lesion (*n* = 1)	Pseudo-arthrosis (*n* = 2)

## Discussion

The clinical results of two revision strategies for failed TDR’s were studied after a mean follow-up of 3.7 and 4.4 years in the removal and fusion group, respectively. In 18 patients, a posterolateral instrumented fusion without removal was performed and in 21 patients removal of the TDR was combined with anterior interbody fusion followed by posterolateral instrumented fusion. The mid-term VAS and ODI scores significantly improved in the removal group compared to pre-revision surgery, while no significant improvement was found in the fusion group. However, the VAS and ODI scores were comparable for both groups at both time points. A clinical successful improvement (≥25 %) in VAS was found in 47 % in the removal group and in 22 % in the fusion group. For ODI, 21 % in the removal group and 27 % in the fusion group showed a clinical successful improvement. An important point to consider is that, in contrast to the fusion only group with no intra-operative complications, the TDR removal group showed substantial complications in nine patients (31 %) during surgery.

The present study was limited by the relatively small number of cases in both groups which may have induced a type II error. In addition, both surgical groups showed heterogeneous patient characteristics. For example, the number of patients who underwent a second stage revision surgery varies considerably.

In the literature, a wide range of complications has been reported in TDR implantation studies. These complications can be divided into (1) treatment related (e.g. pain, wound problems), (2) anterior approach related (e.g. vascular injury, retrograde ejaculation) and (3) prosthesis related (e.g. subsidence, migration) [11]. The number of reoperations varied between 2.3 and 14 % [1, 6, 7, 11, 12, 17]. McAfee et al. [6] reported on 24 patients (9 %) who underwent an anterior TDR revision surgery. Those patients who underwent a revision for failed TDR, all had a suboptimal or poor placement of the TDR. The mean time to revision was 9 months (range 3 days to 34 months). In 4 of these 24 patients (16.7 %), a vascular injury was encountered [6]. In another study, from Leary et al. [17], 18 patients underwent an anterior revision procedure after an average follow-up of 6 months (range 9 days to 4 years). No significant vascular, ureteral and neurological injuries were encountered. However, two patients had a minor left iliac vein injury (11.1 %), and one case of retrograde ejaculation was seen that resolved spontaneously [17]. In our patient group we encountered a vascular injury in 6 out of 29 (20.7 %). To avoid a ureter lesion, we suggest to insert a J-catheter in the left ureter if a left retroperitoneal approach is used. When considering revision surgery it should be realized that removal of the prosthesis has increased risks because of vascular structures and scar tissue [16–18]. The assistance of a vascular surgeon during TDR removal surgery is strongly recommended [16, 17]. Furthermore, the time between TDR implantation and revision may be of importance. In our patients, TDR removal was performed at much larger follow-up as compared to the previously mentioned studies (mean 9.4 years, range 3.1–16.3). When TDR removal is indicated, it should be performed as soon as possible after the initial TDR implantation because of the development of scar tissue and adhesions [17]. Complications such as wound infection and injury to the (superior) hypogastric plexus, which may induce erectile dysfunction and retrograde ejaculation, were not encountered in our series [19].

There is an ongoing discussion about the optimal revision strategy for failed TDRs. In case of an acceptable implant status and position, posterior fusion can be addressed for the treatment of recurrent back pain thought to be facet joint in origin. When the TDR has subsided, migrated or mechanically failed, the pain can be addressed by TDR removal. In our own experience, the results of posterolateral fusion without TDR removal were disappointing in most patients. Therefore, we combined fusion with TDR removal in all cases if the patient accepted the risks of retrieval surgery.

In a previous study, we studied periprosthetic fibrous tissues of the first 16 patients with TDR removal using light microscopy [13]. Results of that study demonstrated the presence of polyethylene wear particles and of periprosthetic inflammatory reactions around a failed TDR in 15 out of 16 patients [13]. We therefore hypothesized that TDR removal will reduce back and leg pain in failed TDRs because the source of wear debris generation is removed, which may diminish inflammatory mediated pain. The present study showed that, although there was no significant difference between the removal and fusion group during post-revision surgery, the VAS score improved significantly in the removal group after 3.1 years. Removal surgery as a second revision strategy in patients who still experience a high amount of pain after posterolateral fusion reduced VAS pain scores non-significantly.

The aim of the present study was to provide mid-term clinical follow-up data on two TDR revision strategies. In agreement with our previous study [15], the VAS and ODI scores showed similar wide ranges, which indicate substantial variability in outcome between the individual patients. Possible explanations for these wide intervals are patient-related factors like number of previous surgeries and number of affected levels. Larger groups will be necessary to assess the effect of patient factors on the success rate of a revision surgery.

In conclusion, the benefit of removing the prosthesis after failed TDR remains unclear. Removal of the TDR may be justified. However, the patient should be counselled about potential risks and complications of this kind of revision surgery which should be carefully weighed up against the possible benefits of TDR removal.
